# Individuals’ willingness to provide geospatial global positioning system (GPS) data from their smartphone during the COVID-19 pandemic

**DOI:** 10.1057/s41599-022-01338-7

**Published:** 2022-09-26

**Authors:** Yulin Hswen, Ulrich Nguemdjo, Elad Yom-Tov, Gregory M Marcus, Bruno Ventelou

**Affiliations:** 1 Department of Epidemiology and Biostatistics, University of California San Francisco, San Francisco, USA; 2 Bakar Computational Health Sciences Institute, University of California San Francisco, San Francisco, USA; 3 Aix Marseille Univ, CNRS, AMSE, Marseille, France; 4 Aix Marseille Univ, LPED, Marseille, France; 5 Microsoft Research Israel, Herzeliya, Israel; 6 Division of Cardiology, University of California San Francisco, San Francisco, USA; 7 ORS-PACA, Marseille, France

**Keywords:** Economics, Science, technology and society, Sociology

## Abstract

This study aims to evaluate people’s willingness to provide their geospatial global positioning system (GPS) data from their smartphones during the COVID-19 pandemic. Based on the self-determination theory, the addition of monetary incentives to encourage data provision may have an adverse effect on spontaneous donation. Therefore, we tested if a crowding-out effect exists between financial and altruistic motivations. Participants were randomized to different frames of motivational messages regarding the provision of their GPS data based on (1) self-interest, (2) pro-social benefit, and (3) monetary compensation. We also sought to examine the use of a negative versus positive valence in the framing of the different armed messages. 1055 participants were recruited from 41 countries with a mean age of 34 years on Amazon Mechanical Turk (MTurk), an online crowdsourcing platform. Participants living in India or in Brazil were more willing to provide their GPS data compared to those living in the United States. No significant differences were seen between positive and negative valence framing messages. Monetary incentives of $5 significantly increased participants’ willingness to provide GPS data. Half of the participants in the self-interest and pro-social arms agreed to provide their GPS data and almost two-thirds of participants were willing to provide their data in exchange for $5. If participants refused the first framing proposal, they were followed up with a “Vickrey auction” (a sealed-bid second-priced auction, SPSBA). An average of $17 bid was accepted in the self-interest condition to provide their GPS data, and the average “bid” of $21 was for the pro-social benefit experimental condition. These results revealed that a crowding-out effect between intrinsic and extrinsic motivations did not take place in our sample of internet users. Framing and incentivization can be used in combination to influence the acquisition of private GPS smartphone data. Financial incentives can increase data provision to a greater degree with no losses on these intrinsic motivations, to fight the COVID-19 pandemic.

## Introduction

The COVID-19 pandemic has highlighted the importance of digital epidemiology based on public health data. (Vuong et al., 2022) Conceptually, the paradox between public health and personal privacy has come into question. In the early stages of the pandemic, public health officials faced critical problems in collecting effective information input from the public. Specifically, the issues around the donation of digital data as it relates to the context of mobile phone privacy became a major roadblock in trying to fight the COVID-19 pandemic. The question of how to effectively influence motivations on the data provided with the sub-question of the influence of monetary, *versus* altruistic motivations, became a key factor in understanding how to resolve the paradox.

One key data source has been human mobility which can be collected from geospatial global positioning system data (GPS) on smartphones that can be used to support efforts to understand the transmission patterns of COVID-19 and to control the effectiveness of public health interventions like contact tracing (Perra, 2021; Beria and Lunkar, 2021; Grantz et al., 2020). GPS data provided by users’ smartphones can be analyzed to obtain a verifiable record of individuals’ human mobility patterns and help predict the future disease trajectory of COVID-19 such as the identification of hotspots and the social and environmental factors that contribute to the further spread of COVID-19.

However, large concerns have been brought up about how users may not readily release their personal data, and if so they will only do it under specific conditions (Acquisti et al., 2016; Posner, 1981). This “privacy concern” (Posner, 1981) must be compensated by an additional means that would drive people to give access to their data. For social scientists, one of the key points in data donation behavior is the motivational mechanism that can be activated to incentivize people to donate their private data. During the COVID-19 pandemic, disclosing personal data can generate social benefits, and so, “data altruism”—data donated for the common good^1^ —may act as a major motivational mechanism. However, “data altruism” by itself may not be able to counteract the strong obstacle of concern for privacy, at least to be able to obtain a sufficient acceptance rate level (estimated at 60%, in Ferretti et al., 2020). Evidence had also emerged in the field of behavioral economic framing (e.g. Oullier et al., 2010), that the form of the message may have an impact, depending on the salience of the social motive for the data donation.

As there is the collective benefit of data donation, the decision involves more than the individual balance between privacy concerns and the participant’s personal willingness to give access to their data. Thus, the public authority may consider another motivational mechanism: a monetary reward, with the aim to trigger people to internalize the collective benefit of their decision. It has already been shown that a monetary reward can bias the self-interest calculation toward data donation decisions (Gefen et al., 2020). However, in the behavioral economic literature, individuals seem to be more sensitive to the loss aversion effort—greater fear of losses than to a symmetric gain. In addition, a strong complication could also come from the “crowding-out effect” of extrinsic incentives. Deci, the founder of the self-determination theory was the first to detect this kind of unexpected effect in professional behaviors (Deci, 1972): workers could do less (rather than more) when they are remunerated for some tasks that they anyway have the intention to do. Economists then progressively developed a reflection on professional motivations (Kreps, 1997) and more generally on prosocial motivations (Frey et al., 1997; Gneezy and Rustichini, 2000). Since the end of the 1990s, several studies on pro-social behavior, particularly in the health domain (either patients or professionals), have confirmed the concerns that monetary payments may crowd out intrinsic and altruistic motives, thus ultimately reducing the overall social contribution (Rothman and Rothman, 2006; Himmelstein et al., 2014). To cite the most famous example, it has been documented that blood donation behavior, initially studied by Titmuss (1971) is above all motivated by a sense of altruism, with a (generally) unanticipated crowding-out effect of monetary incentives (Mellström and Johannesson, 2008): people could give less when they are remunerated for blood donation. In other words, offering a monetary reward may deter users from donating for altruistic means. Thus the purpose of this study is to investigate the willingness to provide digital geospatial GPS data from Smartphones when users are paid *versus* not paid. We also sought to study the reality of a loss aversion phenomenon in the data donation domain by examining the use of a negative versus positive valence in the framing of the message.

Specifically, we aimed to evaluate people’s willingness to provide their geospatial global positioning system (GPS) data from their Smartphone during the COVID-19 pandemic based on different methods of framing and incentives. In this randomized experimental design, we test the suitability of various messages that could increase smartphone users’ willingness to provide their personal information on human mobility.

## Methods

Participants were recruited via *Amazon Mechanical Turk* *(MTurk)*, an online crowdsourcing platform that is one of the suites of *Amazon.com Web Services*. In recent years MTurk had been extensively used in social science research. MTurk enables researchers to recruit participants to perform tasks such as filling out surveys, opinion polls, cognitive psychological studies, and other research services. MTurk rules state that participants can terminate the study by returning the task at any time, without any penalty. Participants on MTurk have a unique Worker ID, which is a semi-random alphanumeric string. Participants’ Worker IDs is associated with the study results making participant *anonymous*, as no identifying information including their names or address can be collected. Additionally, MTurk has several mechanisms in place to protect unauthorized access including protecting the security of information during transmission by using Secure Socket Layer (SSL) software to keep users’ privacy protected.

Users on MTurk have presented a list of potential tasks or Human Intelligence Jobs (referred to as HITs) when they log into their MTurk account. Our research study was listed on MTurk as a HIT and potential participants were proposed a compensation ($0.05—standard compensation to complete a HIT) to complete the HIT questionnaire (5 min). Once users clicked on the HIT they were directed to the online consent which provided further information about the study. If the user agreed to the consent they were directed to complete the study that was hosted on the Qualtrics platform. The first portion of the study questionnaire included questions on demographics, COVID-19 testing history, and whether they know anyone who has tested positive. Inclusion criteria included 18 years or older and owning a smartphone and what type of operating system they used (Android-Google, IOS-Apple).

Users were then randomly assigned to equal arms whereby they were provided messages that related to (1) self-interest; (2) pro-social; (3) monetary motivations for contributing their GPS data from their smartphone to understand the COVID-19 pandemic. Within each arm, participants received either a message framed with either positive or negative valence (creating six questions or “groups” in total) and were asked if they would be willing to contribute their GPS data from their smartphone.

- *Arm 1, self-interest (+valence)*: We will provide you feedback on how to navigate your daily schedule in a safe way with COVID-19.
- *Arm 2, self-interest (−valence)*: We will provide you feedback on if you have been in contact with someone who has tested positive for COVID-19.
- *Arm 3, pro-social (+valence)*: It will help us identify how to re-open your community safely
- *Arm 4, pro-social (−valence)*: It will help us identify hotspots that need to be sheltered in place in your community.
- *Arm 5, monetary (+valence)*: You will receive a $5 bonus payment if you give your GPS data.
- *Arm 6, monetary incentive (−valence)*: You will not receive a $5 bonus payment if you renounce giving your GPS data.

The monetary incentive arm offered a $5 payment for their willingness to provide their GPS data. Participants in this arm that indicated that they were willing to provide their data received a $5 bonus payment, on top of the base $0.05 HIT payment. Assignment to the monetary arm was completely random and equally likely for all participants. The test of the crowding-out effect due to monetary incentives stands in the comparison between arms 5&6 with arms 1–4. The test of the positive vs. negative valence framing effect stands in the comparison of arms 1, 3, and 5 to arms 2, 4, and 6, respectively.

Individuals in all groups that mark that they were not willing to contribute their GPS data received a Vickrey auction. A Vickrey auction or sealed-bid second-price auction (SPSBA) is a type of auction where a “bidder” submits a bid without knowing the bid of the other users in the auction. This auction method has been shown to elicit more truthful values for data provision of personal internet data (Gefen et al., 2020). Thus, in our study, users were asked if they would like to place a “bid” of a selected monetary value of their choosing in exchange for their GPS data. In our study, users were blinded to other users’ bids as well as the maximum threshold bid that was deemed a payout. Here is the sentence they were confronted to

- “You have declined to give your data. Others have refused to be paid $5 to give their location history data. However, we are very interested in capturing your location history data from your Smartphone. We will be asking 1000 people to give their location history data. We will only be paying the people with the lowest 100 bids and bids that are under our threshold.”

Users were able to select a sliding scale of monetary compensation they would take in exchange for their data. The recap of the value of $5 was made to anchor all participants in the same context (including those who were not submitted to the monetary condition at the first step). If they bid higher than our maximum of $10 they were told that their bid was not accepted.

At the end of the survey, participants received a debriefing message to remove their possible deception. They were told that the full objective of this study was to investigate the effectiveness of different messaging in encouraging individuals to contribute their GPS data to COVID-19 and the need to initially withhold some of this information due to the nature of the randomized scheme.

## Economic model

Providing GPS data from a smartphone is an effort for users. The fundamental trade-off is the following: users must decide between their privacy and public safety (the utility of the provision of data, for a public benefit). The economic theory brings the idea that it is possible to reveal the individual utility—or ‘disutility’—associated with an effort by the mean of a proposed monetary compensation (Gefen et al., 2020). Let call $V_{\mathrm {m}}^i$ and $V_{{\mathrm {pros}}}^i$ the valuation of the participant *i* for their effort, under the different experimental conditions (pros for pro-social, m for monetary; the self-interest condition being the baseline does not need a specific subscript), and let us call $V_{{\mathrm {tot}}}^i = V_{\mathrm {m}}^i + V_{{\mathrm {pros}}}^i$ the total value for participant *i* to their data. Let denote $V_{{\mathrm {cost}}}^i$ the cost for the participant *i* in revealing their valuation. The total valuation of participant *P*^*i*^ is given by $P^i = V_{{\mathrm {tot}}}^i - V_{{\mathrm {cost}}}^i$. *P*^*i*^ is the value to be reported if the individual participates in a truth-revealing Vickrey auction. In an experiment where the participant is asked to provide personal data, the decision to provide the data will be taken when *P*^*i*^ would be positive and refused when *P*^*i*^ will be negative.

As highlighted by Gefen et al. (2020), under the assumption that *V*_m_ and *V*_pros_ are independent and that *P* is a linear combination of thereof, all transactions can be represented using a linear model where *P* is the dependent variable and the independent variables are dummies that indicate the type of experimental conditions submitted to the participant. Therefore, each transaction will be represented by the following equation:

$$P^i = V_{\mathrm {m}}^i \cdot x_{\mathrm {m}}^i + V_{{\mathrm {pros}}}^ix_{{\mathrm {pros}}}^i - V_{{\mathrm {cost}}}^i$$

where $x_{\mathrm {m}}^i$ and $x_{{\mathrm {pros}}}^i$ are dummy indicators reflecting the experimental condition that the participant was shown. The linear coefficients obtained as a solution of the econometric equation ($V_{\mathrm {m}}^i$, $V_{{\mathrm {pros}}}^i$) represent the average participant valuation associated with each condition (m, pros). When *P*^*i*^ is not observable, the above linear equation is equivalent to a Probit model estimated on *y*^i*^ where *y*^i*^ is the latent variable of *y*^*i*^, the observable response dummy variable of whether the participant *i* decides to provide his personal data. *y*^*i*^ satisfies *y*^*i*^ = 1 if *P*^*i*^ > 0 and *y*^*i*^ = 0 otherwise.

### Testable assumption

An appropriate framing message can modify the value that subjects attach to their personal data and, therefore, the willingness to transfer their GPS data from their Smartphones, as well as the asked for compensation to do it (*P*^*i*^) when subjects will be confronted with monetary compensation. We test whether:

1/ …experimental conditions change the willingness to provide access to data ($V_{\mathrm {m}}^i \,\ne\, 0$, $V_{{\mathrm {pros}}}^i \,\ne\, 0$)

2/ …experimental conditions change the amount of money requested to provide GPS data (check of $P^i( {V_{{\mathrm {pros}}}^i;\,x_{\mathrm {m}}^i = 0} ) \,\ne\, P^i( {V_{\mathrm {m}}^i;\,x_{{\mathrm {pros}}}^i = 0} )$ when *P*^*i*^ is observed)

Statistical analyses were done using R version 4.11.

## Results

### Descriptive statistics

**Fig. 1:**
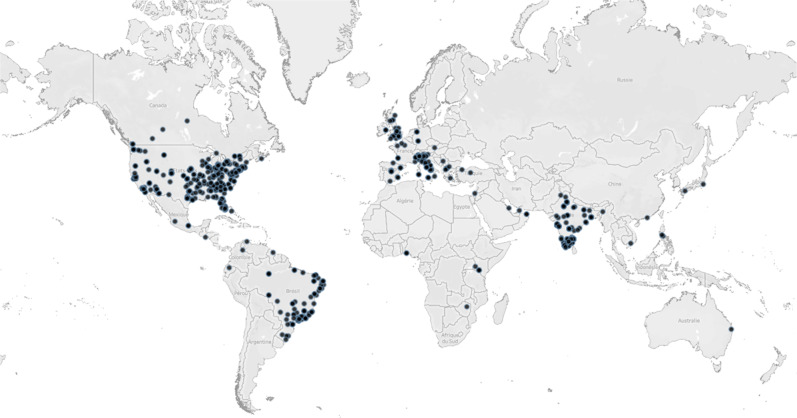
Map of the location of participants: Blue dots represent the regional locations of study participants.

1055 participants were recruited from 41 countries. 445 (42.18%) of them were located in the US, 308 (29.2%) in India, 151 (14.3%) in Europe, 94 (8.9%) in Brazil, and 57 (5.4%) in other countries around the World. The average age of the participants was 34 (s.d. 10) years. Figure 1 gives the map of the location of all the participants. The proportion of males was 57.3% (604) whereas the proportion of females was 42.7% (448), and <0.3% (3) of the participants did not report their gender or have chosen to not disclose it.

1017 (96.4%) participants were owners of a smartphone. 742 (73.01%) of the participants reported using an Android phone operating system whereas 26.98% (274) reported using an IOS operating system. 77.3% (786) of the participants reported knowing somebody who tested positive for COVID-19. (106) 10.48% of the participants reported having a positive COVID-19 status, (416) 40.94% a negative status, (452) 44.46% did not do the test, and (42) 4.11% did not know that status.

Participants were successfully randomized into one of each of the experimental conditions in our study (Table 1, *Χ*^2^-test*, p*-value = 0.48). 329 (32.4%) received the self-interest condition (14.9% arm—1, 17.4% arm 2), 340 (33.4%) received the pro-social condition (16.0% arm 3, 17.4% arm 4), and 348 (34.2%) received the monetary condition (16.3% arm 5, 17.9% arm 6). After receiving one of the 6 experimental conditions, 55.95% (566) of the participants accepted to provide their GPS data (16.42% in the self-interest condition (7.17% arm—1, 9.24% arm—2), 17.60% accepted the pro-social condition (8.45% arm—3, 9.14% arm—4), 21.93% the monetary condition (10.22% arm—5, 11.70% arm—6). Among those who refused one of the six conditions, upon being given the Vickrey auction—told they would receive a monetary incentive for their GPS data, 16.96% (76) accepted to submit a bid.

**Table 1:** Test for equality of proportions in the arms.

Test for equality of proportions without continuity				
Alternative hypothesis: two sided				
	Proportions	*X* ^2^	df	*p*-value
Arm 1	0.1494	4.4566	5	0.4857
Arm 2	0.1748			
Arm 3	0.1602			
Arm 4	0.174			
Arm 5	0.1632			
Arm 6	0.1789			

### Econometric results

For the demographic and health determinants, there was a significant negative association between the type of mobile operating system and participants’ decision to provide their GPS data. Participants using an IOS (Apple) operating system were significantly less willing to provide their GPS data compared to those who use an Android (Google) operating system. Additionally, we found a significant association between the region or country where the participant was located and their willingness to provide their GPS data. Participants living in India or in Brazil were more willing to provide their GPS data compared to those living in the United States. Finally, we found a significant association between the testing COVID-19 status of the participant and their decision to provide their GPS data. Participants who were tested for COVID-19 (positive or negative) were more willing to provide their GPS data. Participants that tested positive for COVID-19 were significantly more likely to provide their GPS data compared to those who tested negative for COVID-19.

The results after receiving one of the six experimental conditions are provided in Table 2. For the first proposal, with all six experimental conditions, we found no significant difference between a positive valence and a negative valence on the willingness to provide a user’s GPS data (columns 1 and 3, Table 2). Based on this result and to increase the number of observations, a second stage model was employed where the conditional arms were grouped into three simplified arms: self-interest (arms 1 + 2), pro-social (arms 3 + 4), and monetary (arms 5 + 6).

**Table 2:** Determinants of the willingness to provide GPS data.

	Dependent variable: willingness to provide GPS data			
	1	2	3	4
*Arm conditions (ref: Negative valence*)				
Positive	−0.061 (0.079)		−0.061 (0.083)	
*Arm conditions (ref: Self-interest*)				
Pro-social benefit		0.047 (0.097)		0.035 (0.102)
Monetary incentive		0.342*** (0.098)		0.310*** (0.102)
*Control variables*				
Gender (ref.: Female)			−0.045 (0.085)	−0.042 (0.085)
Age			−0.006 (0.004)	−0.005 (0.004)
IOS operating system (ref.: Android)			−0.240** (0.100)	−0.227** (0.100)
Know someone with COVID-19 (ref.: No)			0.004 (0.101)	−0.011 (0.101)
*Tested for COVID-19 (ref: Not tested*)				
Positive			0.697*** (0.152)	0.692*** (0.152)
Negative			0.389*** (0.091)	0.383*** (0.091)
Do not know			0.147 (0.220)	0.164 (0.220)
*Location (ref. : US)*				
Brazil			0.411** (0.164)	0.425*** (0.165)
India			0.410*** (0.109)	0.416*** (0.109)
Europe			−0.077 (0.127)	−0.079 (0.127)
*Other countries*			0.108 (0.187)	0.138 (0.188)
Constant	0.179*** (0.054)	0.019 (0.069)	0.093 (0.203)	−0.069 (0.215)
Observations	1017	1017	990	990
Log-Likelihood	−697.416	−690.429	−637.232	−631.840
Akaike Inf, Criteria	1398.833	1386.857	1300.464	1291.681

In this Table 2, we reported the aggregated test for positive *versus* negative valence, with grouped arms (self-interest + prosocial + monetary). We tested also positive *versus* negative valence arm by arm, separately. Tests were not significant.

**p < 0.05; ***p < 0.01.

Columns 2, and 4 of Table 2 show a significant association between the monetary condition (arms 5 + 6) and the decision to provide a user’s GPS data. Based on this result participants are more willing to provide their GPS data if they were told they would receive a $5 monetary compensation.

## Vickrey auction

**Fig. 2:**
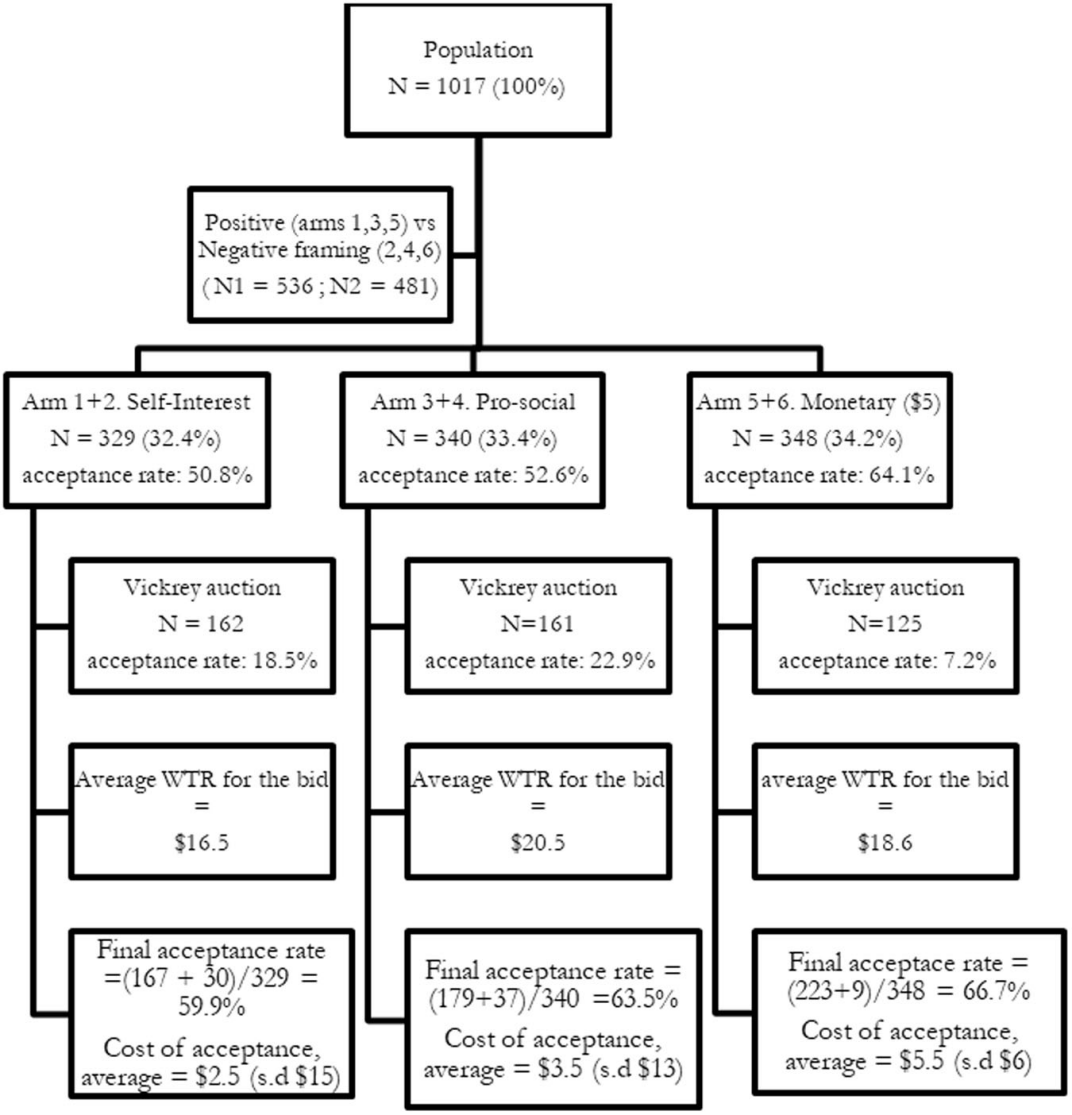
Randomization of three different arms of the study: Chart of participants in each study arm.

**Fig. 3:**
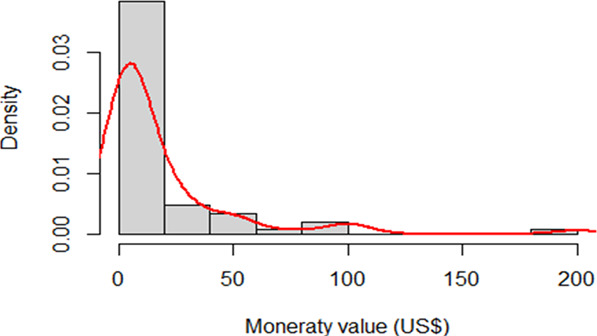
Vickery auction bid value distribution: Monetary bid of participants in exchange for GPS data.

Participants who refused to provide their GPS data after the first request was followed up with a Vickrey auction whereby users were presented with an option to place a monetary bid to be paid for their GPS data, as in Gefen et al. (2020). To analyze the acceptance of this Vickrey Auction (see flow chart (Fig. 2) for a recap of the process), we ran a second step regression on the acceptance of the Vickrey auction process. The results show a significant negative association between the monetary condition (arms 5 + 6) to provide GPS data from their smartphone by using the auction procedure. Thus, among participants who initially refuse to share their GPS data, the type of exposure to the first request influences their decision to bid a price for their GPS data. In addition, Fig. 3 shows the distribution of the monetary bid by the participants who initially (first proposal) refused to provide their GPS data. Those who had received the monetary condition as a first proposal, were less willing to give their data for a bidding price in the Vickrey auction (Table 3).

**Table 3:** Determinants of the acceptance of the Vickrey auction procedure.

	Dependent variable: Follow-up, willingness to provide GPS data	
	1	2
*Arm conditions (ref.: Self-interest)*		
Pro-social benefit	0.156 (0.158)	0.134 (0.168)
Monetary incentive	−0.565*** (0.204)	−0.645*** (0.215)
*Control variables*		
Gender (ref.: Female)		0.066 (0.154)
Age		0.0003 (0.007)
IOS operating system (ref.: Android)		−0.280 (0.179)
Know someone with COVID-19 (ref.: No)		0.238 (0.191)
*Tested for COVID-19 (ref.: Not tested*)		
Positive		0.413 (0.305)
Negative		0.181 (0.160)
Do not know		−4.582 (138.864)
*Location (ref.:* *US)*		
Brazil		0.302 (0.321)
India		−0.023 (0.205)
Europe		−0.088 (0.218)
Other countries		−0.153 (0.360)
Constant	−0.896*** (0.114)	−1.062*** (0.398)
Observations	448	432
Log-likelihood	−196.760	−183.577
Akaike Inf, Criteria	399.520	395.154

Before estimating this second step regression, we first conduct a test for the selection bias using a two steps model, considering a possible self-selection behavior of participants (only those who refused were proposed the auction procedure). The non-significance of the Inverse Mill Ratio (*p*-value of the lambda = 0. 21) suggests that our second-step econometric equation is not biased by the self-selection process at the first proposal.

***p < 0.01.

After refusing to provide their GPS data and being provided with the Vickrey auction to receive monetary compensation in exchange for their GPS data, the average compensation (monetary value) for they will exchange their GPS data in the self-interest experimental condition (arms 1 + 2) is on average *$17* and for the pro-social experimental condition (arms 3 + 4) it is *$21*. If participants received the monetary incentive experimental condition (arms 5 + 6), the average compensation (monetary value) they will exchange for their GPS data *$19*, however, the latter is non-significantly different from 0 (Table 4). The distribution of the monetary value of the GPS data of participants who are confronted with the Vickrey auction procedure (bid price) is presented in Fig. 3.

**Table 4:** Average monetary value of the GPS data among the ‘follow-up proposal’ branch.

	Coefficients	s.e.
*Experimental conditions*		
Self-interest	16.483	(5.988)
Pro-social	20.446	(5.374)
Monetary incentive	18.571	(12.188)
Observations	72	
*R*-square	0261	
Adjusted *R*-square	0229	
Residual std. error	32,245	
*F*-statistic	8.125	

## Discussion

This study was made to assess persons’ willingness to provide their GPS data stored on their smartphones and to better understand how to frame messages that will encourage the provision of such human mobility data. Two specific questions were addressed: (i) the effectiveness of monetary incentives, compared to other motivations and (ii) the effectiveness of a negative versus positive valence in the framing of the message.

Based on our logistic model, our result suggests that framing the use of users’ GPS data as a positive or negative incentive was not significant. A simple positive or negative framing without additional information does not significantly change the approbation rates for giving access to GPS data. In fact, a strong source of privacy behavior arises from incomplete and asymmetric information (Acquisti et al., 2015). It appears that the positive vs. negative valence framing is not enough to push away people’s concern about providing private data on their mobility patterns.

When designing the experimental condition into different types of motivational messages to provide private data, self-interest motivation, pro-social benefit, or monetary benefit, we found that monetizing access to GPS data increased the proportion of participants willing to share their human mobility data. The perspective of a small $5 monetary compensation seems first to generate a higher proportion of acceptance rate (64%). These results parallel a previous study conducted by Munzert et al. (2021) whereby the addition of a small monetary value up to $5 significantly increased the likelihood of users downloading a contact tracing application to fight COVID-19. However, this study did not offer a monetary incentive in the first proposal whereas our study provided a secondary bid proposal, in the form of a Vickrey auction, allowing users to have an additional opportunity to bid for a greater monetary value for their private smartphone data. In our study, offering to indicate a monetary value for their GPS data after the first framing proposal, we found that proposing a payment through an auction mechanism could be also fruitful, with a significant additional increase in acceptance rates, varying between 23% and 7%, depending on the previous framing (self-interest, pro-social), with the monetary arms (5 and 6) having the lower additional acceptance rates at the step of the Vickey option. Munzert et al. (2021) did not evaluate this secondary effect of two monetary incentives and our study gives evidence that individuals who rejected the first monetary incentive are less likely to give up their private data. These findings show that some users estimate their mobility data as “priceless”, as they reject both the first and the second financial incentives; making it clear that their data is not for sale at any price.

When using the result of the elicitation of the monetary value obtained from the bid, the average monetary valuation given by participants was US$17 when they are told their human mobility data will be used for a self-interest purpose. This value is similar to the value given by participants for public use of their internet health data (Gefen et al., 2020). The valuation of participants when the motivational message was the use of their GPS data for a collective purpose was US$21. This valuation, although a little bit higher, is not significantly different from the self-interest condition. At the end of the survey (taking into account that spontaneous data donation exists, for free), we evaluate the average cost of acceptance, per sharing accepted, US$3 for the self-interest framing to US$4 for the pro-social benefit framing, and US$6 for the monetary incentive framing. This means that, in return for a small compensation to users, it is possible for a service provider to obtain the needed GPS data. In addition, the significant difference between these average costs (*p*-value = 0.0104) suggests that an efficient approach to encourage smartphone users to provide their historical location data is to start with a self-interest incentive framing for the request of private smartphone data, and, for those who remain resistant, that a follow-up of a small monetary incentive will be effective. However, certain individuals, who initially rejected to provide their data, may still reject both the first framing messages and a secondary monetary incentive.

From a theoretical point of view, the findings of the study tend to invalidate the preconception of a crowding-out effect, at least for the specific domain of data donation behavior. On the contrary, the financial motivation appears to be “crowding-in”, since the arms (5 and 6), with a monetary reward, are tested much more fruitful in terms of average adhesion to personal data access. We rather validate previous research on monetary incentives for the adoption of contact-tracing apps (Munzert et al., 2021) and the general statement that crowding theory can be either “in” or “out”, depending on the precise context (Frey et al., 2012; Gneezy et al., 2011). Our results build on these previous researches as we demonstrate that there is no crowding-out effect between intrinsic and extrinsic motivations for the provision of private smartphone data. Methods of framing and of incentivization can be used in conjunction to influence the user’s willingness to provide their private human mobility data from their smartphone. No loss for the public authority was found when a mixed strategy was selected. On the contrary, this dual strategy had the best cost-effectiveness ratio to incentivize data provision. Thus the study’s findings have the ability to contribute to lowering the social cost of epidemic control by informing evidence-based policymaking efficiently (Vuong, 2018).

Lastly, our results indicate that participants who knew their COVID-19 serology status are more willing to share their GPS data than those who have not been tested. The same was seen for participants who knew someone who had COVID-19. This may indicate that an “empathic” response was taking place whereby if participants have a personal health experience with the disease of study, they are more likely to exhibit altruistic or pro-social behaviors which have also been seen in previous data donation studies (Gefen et al., 2020; Vuong and Napier, 2015; Papachristou and Whitcomb, 2004). A theoretical explanation could come from the “mindsponge mechanism” which elucidates how and why an individual observes and ejects cultural values depending on the external setting (Vuong and Napier, 2015); in our study, this is shown through having been tested for COVID-19 or knowing someone positive. Our results also suggest that participants living in Brazil or India are more willing to provide their mobile phone GPS data compare to those living in the US. The latter result may reflect a local context behavior (Eggo et al., 2021). Indeed, during the period of study, those two countries (Brazil and India) were some of the countries with the highest prevalence rate of COVID-19, again perhaps stimulating a pro-social mindset (Vuong and Napier, 2015) and, then, eliciting an empathetic response. This may imply that greater experience with COVID-19 either through testing or through vicarious (indirect) community experiences moderated participants’ fears of privacy and increased their altruistic behavior toward public safety (Pomery et al. 2009). Finally, we found that the type of operating system of the user’s smartphone may play a role in the willingness to provide location data. Users of the IOS (Apple) operating system are less willing to share their mobile phone GPS data compare to users of the Android (Google) operating system which may indicate a selection bias that those who opt to use an IOS system have greater privacy concerns than the others.

This paper has some limitations that should be recognized. One of the first is the conceivable sensitivity of our results to the population surveyed. Recruitment on Amazon Mechanical Turk permits a large and costless sample but may create biases. Tests on the reliability, of behavioral research, of data collected through the Amazon-Turk platform, are reassuring (Crump et al., 2013; Arechar et al., 2018). However, they may be insufficient to be sure that in part the response we have in our experiment was not biased by the profile of the Amazon-Turk users, who may be more likely to accept data transfers (in other words, the sample members of this platform are self-selected and might be in general more willing to let access to their data than the general population). Note that, during the COVID-19 pandemic, this method of data collection was a necessary measure, as lockdowns prohibited in-person data collection. The RCT design was also assuring that the observed differences between arms were not biased in relative terms (measurement of the effect), although the absolute rates could be -as mentioned, due to the specific population recruited on platforms. Another limitation may lie in the way we introduced and studied the loss-aversion effect. Generally, this has to be associated with a first “endowment”, which, in turn, allows to put people in a lost context. This was not possible for arms 1–4 (self-interest/pro-social), so for a reason of parallelism, we did not do it for the monetary arms (5 and 6).

## Conclusion

Country of origin and COVID-19 testing status influences the behavioral response to sharing private GPS smartphone data. Self-interest and pro-social motivations for data donation are sufficient to encourage donation if the target is around a 50% acceptance rate. However, supplementing prior intrinsic motivations with monetary incentives regarding the provision of private human mobility mobile data has an additive effect on influencing users’ decisions, which could help to reach the 60% acceptance rate level needed to fight the epidemic efficiently. Communications that promote altruistic donation with the addition of financial compensation will encourage the most active participation of users to provide private location data.

## Data Availability

The datasets generated during and analyzed during the current study are not publicly available due to issues of confidentiality and privacy. Data may be available from the corresponding author upon reasonable request.

## Footnotes

1 In this Act, the commission aimed at fostering populations to trust in the facilitation of voluntary data disclosure: *Official Journal of the European Union*, 560th plenary session of the European Economic and Social Committee (JDE)—INTERACTIO, 27.4.2021–28.4.2021, part III Preparatory acts European Economic and Social Committee. 16.7.2021.
