# Correction: Individuals’ willingness to provide geospatial global positioning system (GPS) data from their smartphone during the COVID-19 pandemic

**DOI:** 10.1057/s41599-022-01465-1

**Published:** 2022-12-19

**Authors:** Yulin Hswen, Ulrich Nguemdjo, Elad Yom-Tov, Gregory M Marcus, Bruno Ventelou

**Affiliations:** 1 Department of Epidemiology and Biostatistics, University of California San Francisco, San Francisco, USA; 2 Bakar Computational Health Sciences Institute, University of California San Francisco, San Francisco, USA; 3 Aix Marseille Univ, CNRS, AMSE, Marseille, France; 4 Aix Marseille Univ, LPED, Marseille, France; 5 Microsoft Research Israel, Herzeliya, Israel; 6 Division of Cardiology, University of California San Francisco, San Francisco, USA; 7 ORS-PACA, Marseille, France

**Keywords:** Economics, Science, technology and society, Sociology

Correction to: *Humanities and Social Sciences Communications* 10.1057/s41599-022-01338-7, published online 26 September 2022.

In this article the funding from the French government under the “France 2030” investment plan managed by the French National Research Agency (reference: ANR-17-EURE-0020) and from Excellence Initiative of Aix-Marseille University - A*MIDEX.” and Federal funds from the National Institutes of Health, Department of Health and Human Services, under Contract No. 75N91020C00039 was omitted. The original article has been corrected.

